# Sub-bandgap near-infrared photovoltaic response in Au/Al_2_O_3_/n-Si metal–insulator–semiconductor structure by plasmon-enhanced internal photoemission

**DOI:** 10.1186/s11671-023-03818-4

**Published:** 2023-03-07

**Authors:** Xiyuan Dai, Li Wu, Liang Yu, Zhiyuan Yu, Fengyang Ma, Yuchen Zhang, Yanru Yang, Jian Sun, Ming Lu

**Affiliations:** 1 Department of Optical Science and Engineering, and Shanghai Ultra-Precision Optical Manufacturing Engineering Center, Fudan University, Shanghai, 200433 China; 2 Yiwu Research Institute of Fudan University, Yiwu, 322000 Zhejiang China

**Keywords:** Sub-bandgap NIR PV response, Surface plasmon, Internal photoemission, Metal–insulator–semiconductor, Field-effect passivation

## Abstract

Silicon sub-bandgap near-infrared (NIR) (λ > 1100 nm) photovoltaic (PV) response by plasmon-enhanced internal photoemission was investigated. The Si sub-bandgap NIR PV response, which remains unexploited in Schottky junction-like solar cell device, was examined using nanometer sized Au/Al_2_O_3_/n-Si junction arrays. This kind of metal–insulator–semiconductor structure was similar in functionality to Schottky junction in NIR absorption, photo-induced charge separation and collection. It showed that NIR absorption increased steadily with increasing volume of Au nanoparticles (NPs) till a saturation was reached. Simulation results indicated the formation of localized surface plasmon on the surfaces of Au NPs, which was correlated well with the observed NIR absorption. On the other hand, the NIR PV response was found sensitive to the amount and size of Au NPs and thickness of Al_2_O_3_. Chemical and field-effect passivation of n-Si by using Al_2_O_3_ and SiO_2_ were used to optimize the NIR PV response. In the current configuration, the best PV conversion efficiency was 0.034% at* λ* = 1319 nm under illumination power of 0.1 W/cm^2^.

**Supplementary Information:**

The online version contains supplementary material available at 10.1186/s11671-023-03818-4.

## Introduction

Today’s low-carbon economy demands more efficient renewable energy sources. At present, Si solar cells dominate the photovoltaic (PV) market [1, 2]. Significant advances have been made in promoting the photoelectric conversion efficiency of Si solar cell in the past decade [3, 4]. Typical examples include perovskite/Si tandem solar cells with large open-circuit voltage and TOPCon Si ones with large short-circuit current density and fill factor [5–8]. Although the photoelectric conversion efficiency of Si solar cell is approaching the Shockley–Queisser limit of 30% for single junction Si solar cell [9], the current methodology to improve the cell performance is still limited to the reduction of electrical loss as well as optical loss mainly in the wavelength range of < 1100 nm, corresponding to photon energy higher than Si bandgap of 1.1 eV. The solar radiation in the Si sub-bandgap near infrared (NIR) region has not been fully utilized for PV applications. How to extend the PV response to the Si sub-bandgap infrared region is therefore well worth investigation to further improve the Si PV performance and overcome the Shockley–Queisser limit. So far, various efforts have been made to up-convert NIR photons into visible ones [10–14]. Nonetheless, the intrinsic low efficiency of non-linear process of up-conversion has made it unpromising for utilization of NIR in photovoltaics [12, 13]. On the other hand, it has been found that the structure of metal semiconductor contact, or Schottky junction, has great potential in expanding the responsive spectra range of solar cells, because the Schottky barrier is usually lower than the semiconductor bandgap [15, 16]. By forming Schottky junction, the hot carriers, generated in metallic nanostructure through non-radiatively decay of excited surface plasmons [16, 17], can be extracted by semiconductor via plasmon-enhanced internal photoemission (IPE) before thermal relaxation [18–21]. However, relevant researches mainly focus on TiO_2_ cells (bandgap of 3.2 eV), reporting power conversion efficiencies of 0.02–0.03% working in visible wavelength range [22, 23]. Although these reported efficiencies are seemingly low, they demonstrated a breakthrough in material bandgap limitation and provided beneficial inspiration to study the Si counterpart. In fact, Schottky junctions as well as metal–insulator–semiconductor (MIS) ones have been used in NIR photo-detection (PD) [19, 24–28]. Considering that PD and PV devices are similar in working process and device structure, in this work, we investigated the Si sub-bandgap NIR PV response of nanometer-sized Au/Al_2_O_3_/n-Si MIS junction arrays. It is found that the nano-MIS junction arrays not only absorb the NIR light, but also offer built-in electric field to separate photo-induced charges, via a process of IPE, which is enhanced by localized surface plasmons (LSPs) on Au nanoparticles (NPs).

## Experimental

### Preparation of textured Si substrate

A double-side polished n-type (100) Si wafer (1 ~ 10 Ω∙cm, 200 ± 10 μm thick) was used as the substrate. Firstly, the Si wafer was ultrasonically cleaned in acetone, ethanol and deionized water consecutively, and then dried by nitrogen. To fabricate textured Si, the Si wafer was immersed in an etching solution of NaOH (2 wt%), Na_2_SiO_3_ (2 wt%) and isopropyl alcohol (7 vol%) at 80 °C for 20 min. Then it was rinsed in deionized water and cleaned in solution of H_2_O:H_2_O_2_:NH_3_ = 6:1:1 at 70 °C for 20 min and H_2_O:H_2_O_2_:HCl = 6:1:1 at 70 °C for 10 min consecutively. Finally, the wafer was dipped in dilute HF (1%) for 1 min.

### Preparation of solar cell device

An Al_2_O_3_ layer was deposited on the textured Si by magnetron sputtering and then annealed in a forming gas of hydrogen and nitrogen (H_2_:N_2_ = 10%:90% in volume) at 350 °C for 20 min to passivate the front surface of the Si substrate. Au was then deposited on Al_2_O_3_ at a rate of 5 nm/min by pulsed laser deposition with a frequency-doubled Q-switched Nd:YAG laser at a base pressure lower than $2\times {10}^{-4}$ Pa. The laser worked at a constant repetition rate of 10 Hz and the laser fluence on Au target was about 1.0 J·cm^−2^. Subsequently, the deposited Au thin film was annealed in a forming gas of hydrogen and nitrogen (H_2_:N_2_ = 5%:95% in volume) at 450 °C for 30 min to form Au NPs. An 80.0-nm-thick indium-tin-oxide (ITO) layer and ~ 1.0 μm thick Ag grid were deposited as the front electrode. A 20.0-nm thick SiO_2_ layer for surface passivation was deposited onto the backside of the textured Si by electron beam evaporation, and a 1.0 μm thick Al layer was grown by resistance heating as the rear electrode. Finally, thermal annealing was conducted at 450 °C in nitrogen for 5 min to form Ohmic contacts. The area of solar cell device was 1 $\times$ 1 cm.

### Characterization

The surface morphology and components of the Schottky junction were measured with scanning electron microscopy (SEM, Philips, XL30) and energy dispersive X-ray spectroscopy (EDS, Aztec X-MaxN 80, Oxford Instruments), respectively. The absorption spectra were obtained by an NIR spectrometer (Ideaoptics, NIR2500) with an integrating sphere. External quantum efficiency (EQE) of the solar cell was measured by a QE/IPCE system of Oriel/Newport. The current density–voltage characteristics of solar cells were measured under NIR light illumination using a source meter (Keithley, SMU2400). The NIR light source was a 1319 nm laser diode (CNI laser, MIL-H-1319).

## Results and discussion

**Fig. 1:**
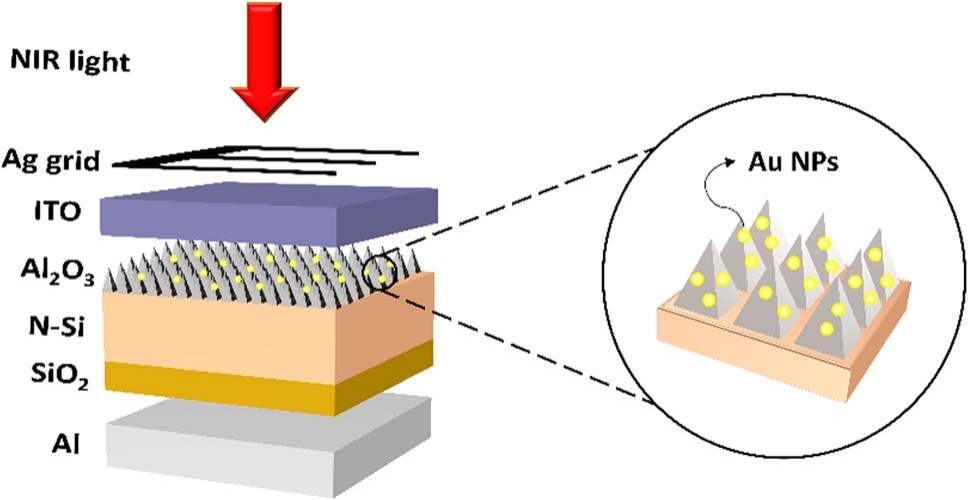
Schematic of textured-Si/Al_2_O_3_/Au nano-MIS Schottky NIR solar cell

Figure 1 shows a schematic of the prepared nano-MIS NIR PV device. The front side of the cell faced a 1319 nm light beam with illumination power of 0.1 W/cm^2^. The textured Si enhanced the light absorption and helped the formation of nano-MIS junctions for NIR absorption at the meantime. The SiO_2_ and Al_2_O_3_ were passivation layers that saturated the dangling bonds on the Si surface so that trapping of photo-induced charges by these defects could be diminished [29–33]. Meanwhile, they also provided electric fields at the interface of oxide and Si, which could facilitate charge separation of photo-induced carriers [34].

**Fig. 2:**
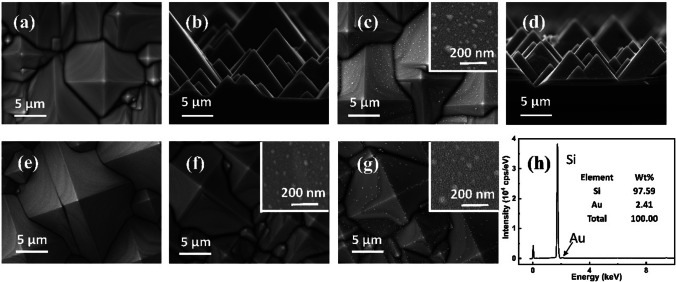
**a–c** Top-view and **b–d** side-view SEM images of the textured Si and textured Si with Au NPs formed from 25 nm Au film annealing. **e** The deposited 25 nm Au film before annealing. Au NPs formed after annealing with different sizes for the apparent thicknesses of **f** 10 nm and **g** 50 nm. The insets are enlarged images for different sizes of Au NPs. **h** EDS spectrum of the Si/Au Schottky junction

The top- and side-view SEM images of the textured Si are given in Figs. 2a, b, respectively. It featured micrometer sized pyramid-like structures. The average width of the Si pyramid base was $6.8\pm 1.5$ μm, and the average height was $5.0\pm 0.9$ μm. Figures 2c, d show the corresponding top- and side-view images for textured Si with Au NPs formed by Au film annealing. The Au deposition time was 5 min, which was equivalent to an apparent thickness of 25 nm. The inset shows an enlarged SEM image, and the average size of Au NPs was $36.2\pm 4.8$ nm. Figure 2e shows the 25 nm Au film before annealing. The Au film was flat and no Au NPs had been formed. Different sizes and distributions of Au NPs exhibited in Figs. 2f, g were formed by annealing Au films with apparent thicknesses of 10 and 50 nm, respectively. The corresponding average diameters of Au NPs were $24.1\pm 1.8$ and $53.6\pm 4.3$ nm, respectively. The average size of Au NPs increased with the growing thickness of the deposited Au film, which is consistent with the relevant studies [35]. For case of 50 nm Au, a small amount of obviously larger Au islands existed on the ridges of Si pyramids that could be more favorite for nucleation of Au NPs. Figure 2h gives the EDS spectrum of Si/Au sample in Fig. 2c. Signals of Si and Au were observed as expected.

**Fig. 3:**
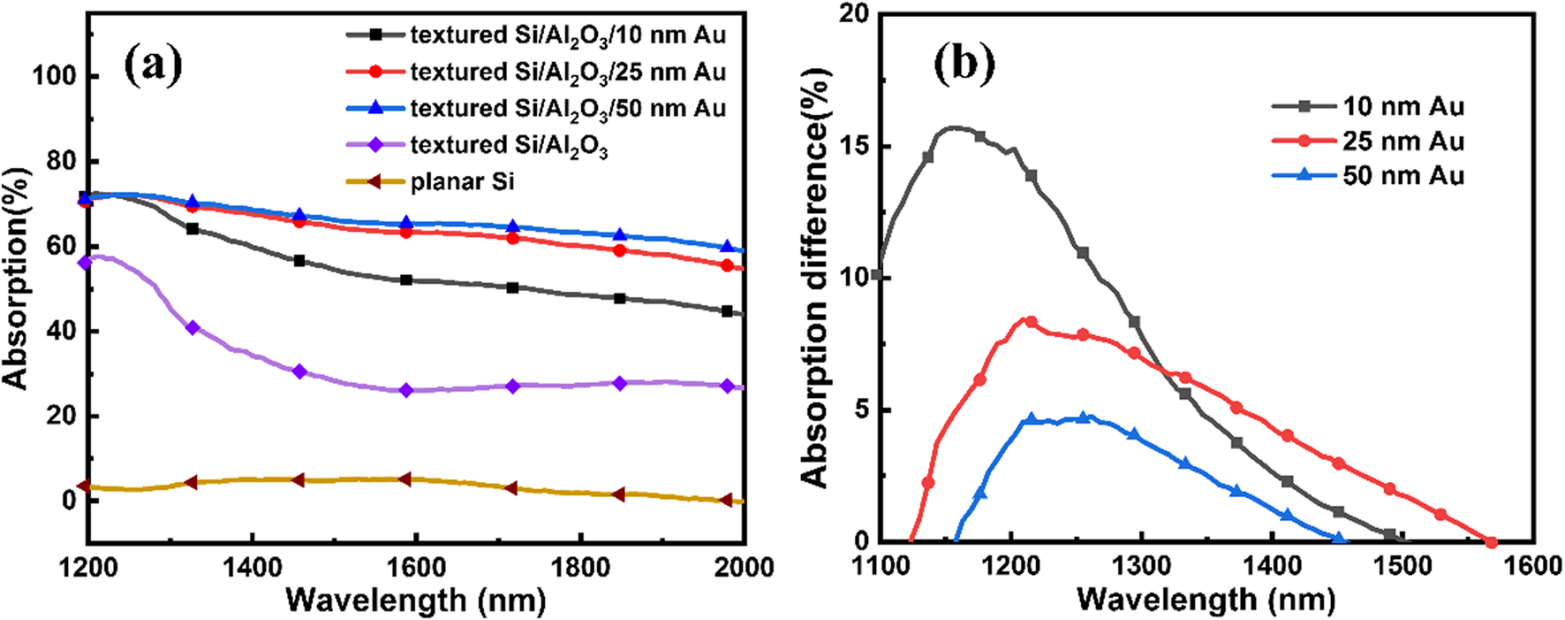
**a** The absorption spectra for planar Si, textured Si/Al_2_O_3_ and nano-MIS junctions with Au NPs formed by different apparent thicknesses. Al_2_O_3_ thickness was 1 nm. **b** The absorption difference spectra between the nano-MIS structures with Au NPs formed by annealing and the structures with the same apparent thickness Au without annealing

Figure 3a shows the absorption spectra for planar and textured Si and for different nano-MIS junctions. The NIR absorption of planar Si could be ascribed to the defect states induced by n-type doping and free carrier absorption [36]. After fabrication of textured Si, the NIR absorption arising from dopant energy levels was improved considerably, indicating the availability of light trapping by the pyramid micro-structures [37–39]. With the deposition of Au and the formation of nano-MIS junctions, the NIR absorption was enhanced considerably due to IPE mechanism [19, 40–42]. From 10 to 50 nm apparent thicknesses of deposited Au, the absorption increased steadily with the growing thickness of Au and reached saturation. The NIR absorption enhancement for larger Au deposition thickness could be attributed to the increasing area of nano-MIS junctions, as shown in Figs. 2c, f and g. On the other hand, localized surface plasmons (LSPs) induced by the incident NIR light on Au NPs would also contribute to the NIR absorption [22, 43–45]. To demonstrate the role of incorporated Au NPs in NIR absorption, Fig. 3b gives the absorption difference spectra between the nano-MIS structures with Au NPs and the structures with corresponding Au film before annealing. A clear trend of further NIR absorption enhancement by formation of Au NPs and LSPs was thus seen. The absorption difference spectra in Fig. 3b showed resonant absorption peaks in 1100–1600 nm NIR wavelength range, which were induced by the excitation of LSPs [46, 47]. Identical Au nanoparticles periodically distributed on a silicon substrate would show resonance absorption peaks in NIR wavelength, while in a fabricated sample, nanoparticles with various sizes would result in broadband absorption enhancement [48]. By subtracting the absorption of corresponding Au film structure without LSPs from the broadband absorption in Fig. 3a, the resulting Fig. 3b could be regarded as a representative or effective resonance absorption peak for fabricated samples with Au NPs, which exhibited the strength and wavelength of LSP qualitatively [24, 48]. The resonance peak wavelength experienced a red-shift from 1156 to 1262 nm as the Au NP size increases from 24.1 nm (corresponding to 10 nm Au) to larger values formed by 50 nm Au, similar to the red-shift in the literature [42, 48]. It is seen that with the increasing deposition of Au, the strength of LSP became weaker, indicating that more Au thin film were grown.

**Fig. 4:**
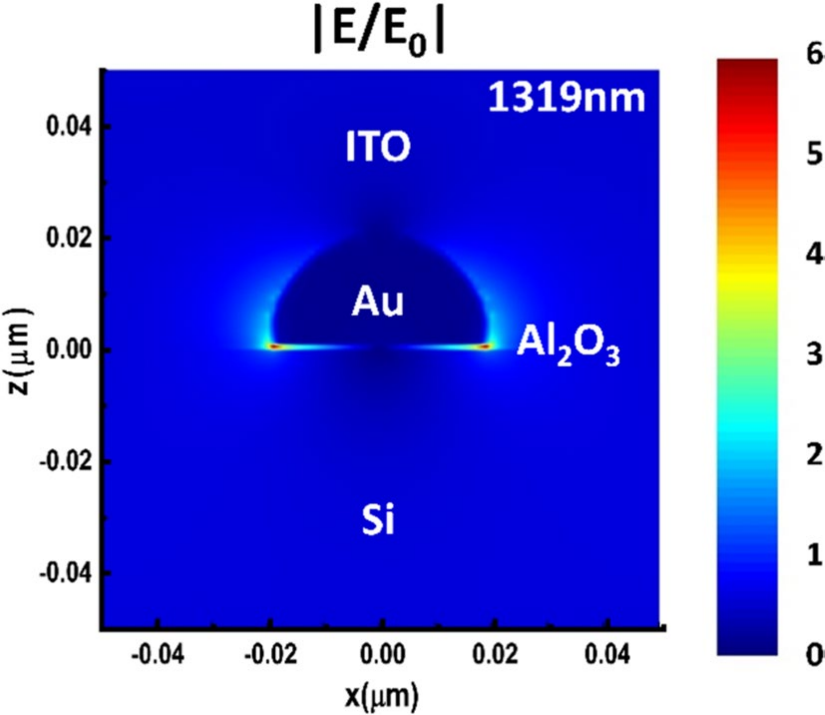
Electric field distribution of Au NP sandwiched by Al_2_O_3_, planar Si and ITO under 1319 nm illumination, simulated by FDTD

To elucidate the LSP-enhanced inner photoemission, electric field distribution was calculated by finite-difference time-domain (FDTD) simulation, as shown in Fig. 4. A simulated distribution of LSP electric field around a half-sphere Au NP surrounded by 1 nm Al_2_O_3_, Si and 80-nm thick ITO film was displayed, under the illumination of 1319 nm incident light. The electric field intensity (E) is re-scaled by the incident field intensity (E_0_). For simplicity, the radius of Au NP was set at 20 nm, and the NP was placed on the surface of Al_2_O_3_ and planar Si. It is seen that bright spots existed mainly at the bottom of the Au NP near the boundary between Au NP and Si, which showed enhancement of electric field. The LSP electric field increased the kinetic energy of hot electrons [45], and helped to overcome the MIS barrier, thus increasing the photo-induced current.

**Fig. 5:**
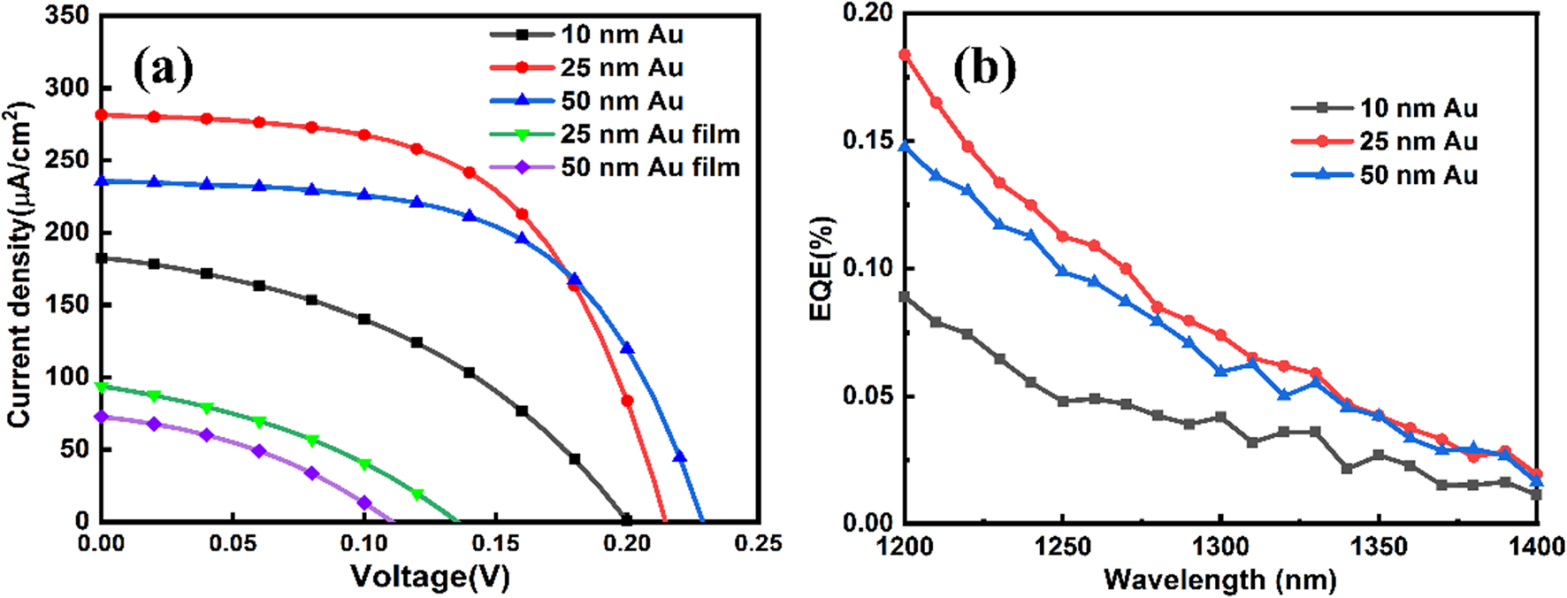
**a** Current density–voltage curves of textured-Si/Al_2_O_3_/Au MIS Schottky solar cell under the illumination of 1319 nm NIR light. **b** EQE of the nano-MIS solar cell with different sizes of Au NPs fabricated by annealing 10 nm, 25 nm and 50 nm Au in 1200–1400 nm

Figure 5a shows the PV response of current density–voltage curves of the MIS PV devices at 1319 nm with illumination power of 0.1 W/cm^2^. Table 1 lists the measured PV parameters of short-circuit current density (J_SC_), open-circuit voltage (V_OC_), fill factor (FF) and conversion efficiency (η). Results for the devices with Au film not annealed were also exhibited, referred to as 25 nm Au film and 50 nm Au film. Such devices had explicitly lower J_SC_ and V_OC_ and worse performance, compared with devices with Au NPs, demonstrating the importance of LSP in sub-bandgap NIR photovoltaic response [49, 50]. Moreover, for ITO/Al_2_O_3_/n-Si structure device without Au NPs, almost no photovoltaic response was found, indicating that Au NPs were the main absorber and determinant factor for photoresponse of 1319 nm light (Fig. S1). The performance of the devices of nano-MIS junctions with Au NPs did not evolve in a similar way as the NIR absorption shown in Fig. 3a. The energy conversion efficiency was the largest for the case of 25 nm Au. This is because that the strength of LSP decreased with the increasing Au deposition time, hence the enhancement of inner photoemission by LSP was weaker, as a result, a compromise of conversion efficiency appeared for the case of 25 nm. Another reason could be due to the difference in the so-called mean free path of hot electron at Au NPs. It is noticed that the average radii of Au NPs formed at 10, 25 and 50 nm apparent thicknesses were 12.0, 18.1 and 26.8 nm, respectively. The mean free path of hot electron at Au NP was ~ 23 nm in length [51–53], which defines a mean distance that a photo-induced hot electron could move freely on the Au NP surface before being thermally dissipated. The average size of Au NPs formed by 50 nm apparent thickness was larger than the mean free path, while that for 25 nm was within the range. Since that the NIR light was incident directly onto the nano-MIS junctions as shown in Fig. 1, hot electrons appeared at the Au NP surface region away from the MIS junctions. Therefore, hot electrons were more difficult to reach and tunnel through the junctions for the case of 50 nm Au compared to the 25 nm one. Since the NIR absorption of nano-MIS structure for 10 nm Au was evidently lower as shown in Fig. 3a, the J_SC_ of corresponding device had lower value than the one of 25 nm and 50 nm Au. When the apparent thickness of Au was raised from 10 to 50 nm, the V_OC_ increased gradually from 0.20 to 0.22 V. This could be explained by the increasing area of nano-MIS junction [54]. For an ideal n-type Si/Au Schottky solar cell under AM0 illumination, the V_OC_ was ~ 0.3 V [55], therefore, it is acceptable that the V_OC_ of the nano-MIS solar cell here was ~ 0.2 V under NIR illumination. Figure 5b displays the EQE of devices with different sizes of Au NPs in sub-bandgap NIR wavelength range. With the increasing size of Au NPs, the variation of EQE was consistent with the changing trend of J_SC_ tested under 1319 nm illumination.

**Table 1:** Photovoltaic parameters of nano-MIS Schottky solar cells with different Au apparent thickness and devices with corresponding Au film

Samples	J_SC_(mA/cm^2^)	V_OC_(V)	FF(%)	$\upeta$(%)
10 nm Au	0.183	0.20	40.7	0.015
25 nm Au	0.282	0.21	58.2	0.034
50 nm Au	0.235	0.22	60.4	0.031
25 nm Au film	0.094	0.14	34.7	0.005
50 nm Au film	0.073	0.11	36.8	0.003

**Fig. 6:**
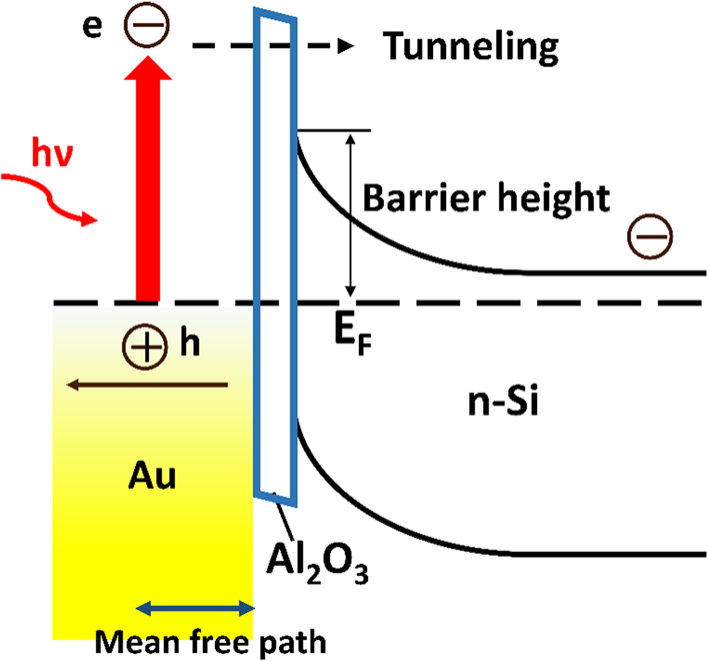
Energy diagram of textured Si/Al_2_O_3_/Au nano-MIS Schottky junction solar cell

Figure 6 shows the schematic energy diagram of the nano-MIS NIR PV device. Since fixed positive and negative charges existed at the interfaces between SiO_2_/Si and Al_2_O_3_/Si, respectively [30, 32], they strengthened the energy band bending of the MIS junction region, and facilitated the photo-induced charge transport. According to the IPE mechanism, hot electrons were excited from Au NPs and entered the conduction band of Si by tunneling through the Al_2_O_3_ layer [15, 19]. They were then pushed toward the rear electrode of Al by the built-in electric field. At the meantime, the holes generated in Au NPs were pushed toward the front electrode of ITO and Ag grid [42].

**Fig. 7:**
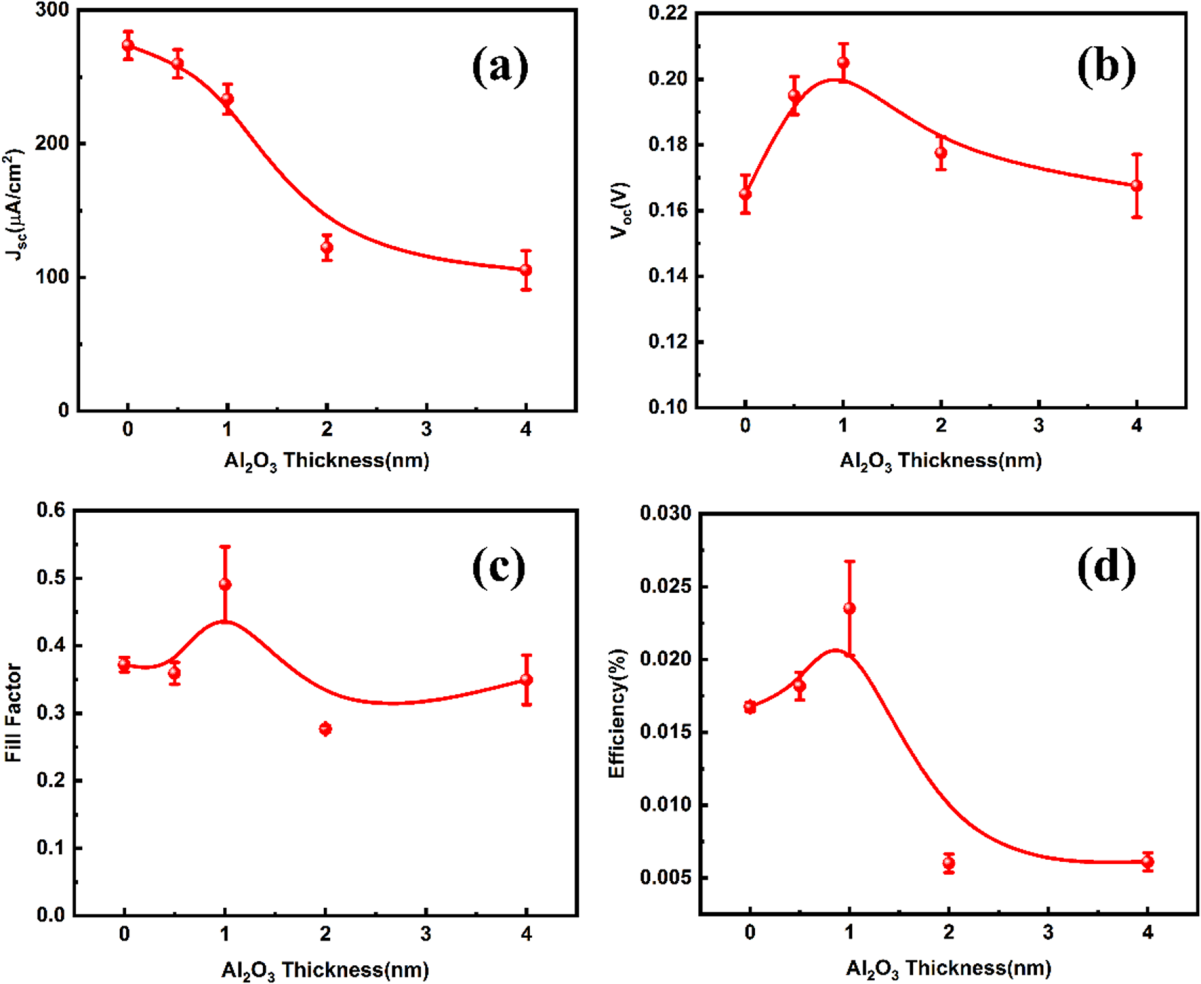
Solar cell parameters as a function of Al_2_O_3_ thickness for nano-MIS device with Au apparent thickness of 25 nm. **a** Short circuit current density, **b** open circuit voltage, **c** FF, and **d** conversion efficiency. Error bars represent the standard deviation and the average is calculated from at least four similar devices

To examine the effect of Al_2_O_3_ layer on the PV parameters, Figs. 7a–d plot the short-circuit current density, open-circuit voltage, fill factor and conversion efficiency as functions of the layer thickness, respectively. From Fig. 7a, it is seen that the J_SC_ decreased all the way with the increasing thickness. To fulfill the separation of photo-induced charges, hot electrons should tunnel through the MIS barrier as indicated in Fig. 6. Therefore, the photo-induced current would depend on the barrier width in a decay manner. Figure 7a suggested this trend. When Al_2_O_3_ thickness was within 1 nm, the passivation of interface defects helps to improve the J_SC_, thus slowing down the decaying trend. The J_SC_ followed an exponential dependence law with barrier thickness for cases beyond 1 nm, which is in agreement with the mechanism of Fowler–Nordheim field-assisted transport [22]. For the evolution of V_OC_, it increased at first till a maximum, and then decreased. It is known that fixed negative charges existed at the interface between Al_2_O_3_ and Si [32]. This kind of field-effect passivation strengthened the band bending at the MIS junction, which tended to increase the V_OC_. However, when the Al_2_O_3_ layer thickness further increased, the number of fixed negative charge approached saturation, but the J_SC_ decreased significantly as shown in Fig. 7a, leading to decrease in the voltage [56]. Another explanation for the improved V_OC_ by proper Al_2_O_3_ thickness could be associated with the increment of Schottky barrier height (${\phi }_{b}$). The Schottky barrier height was 0.77 eV and 0.81 eV for Au/n-Si and nano-MIS structure with 1 nm Al_2_O_3_, respectively, which was obtained by fitting of square root of EQE versus photon energy in 1200–1400 nm wavelength range [57]. For Schottky diode, V_OC_ is linearly proportional to the Schottky barrier height [58]. Within 1 nm Al_2_O_3_ thickness, the V_OC_ of device increased in accordance with the barrier height (Fig. S2). The ${\phi }_{b}$ value increased by 0.04 eV, consistent with the average improvement (~ 0.04 V) between the two devices. From I–V curves of two devices under dark condition (Fig. S3), it could be noticed that the saturation current at reverse bias was evidently lowered after introducing 1 nm Al_2_O_3_, which verified the role of Al_2_O_3_ to raise the Schottky barrier height. Figure 7c gives the dependence of fill factor on Al_2_O_3_ thickness. The FF is mainly related to the sheet resistance of these devices [22]. Since the sufficient passivation of dangling bond defects at Au/Si interface could decrease sheet resistance, FF reached maximum when Al_2_O_3_ thickness was 1 nm. For larger values of thickness, FF evidently decreased due to increasing sheet resistance of thicker Al_2_O_3_ film. From Fig. 7d, it is seen that an optimal thickness of Al_2_O_3_ existed in this nano-MIS device in terms of conversion efficiency. The average conversion efficiency of 1319 nm light was highest for 1 nm Al_2_O_3_, and the best efficiency of 0.034% has been experimentally found with PV parameters shown in Table 1. Besides, the role of SiO_2_ layer on the rear surface of device was mentioned in Fig. S4.

**Fig. 8:**
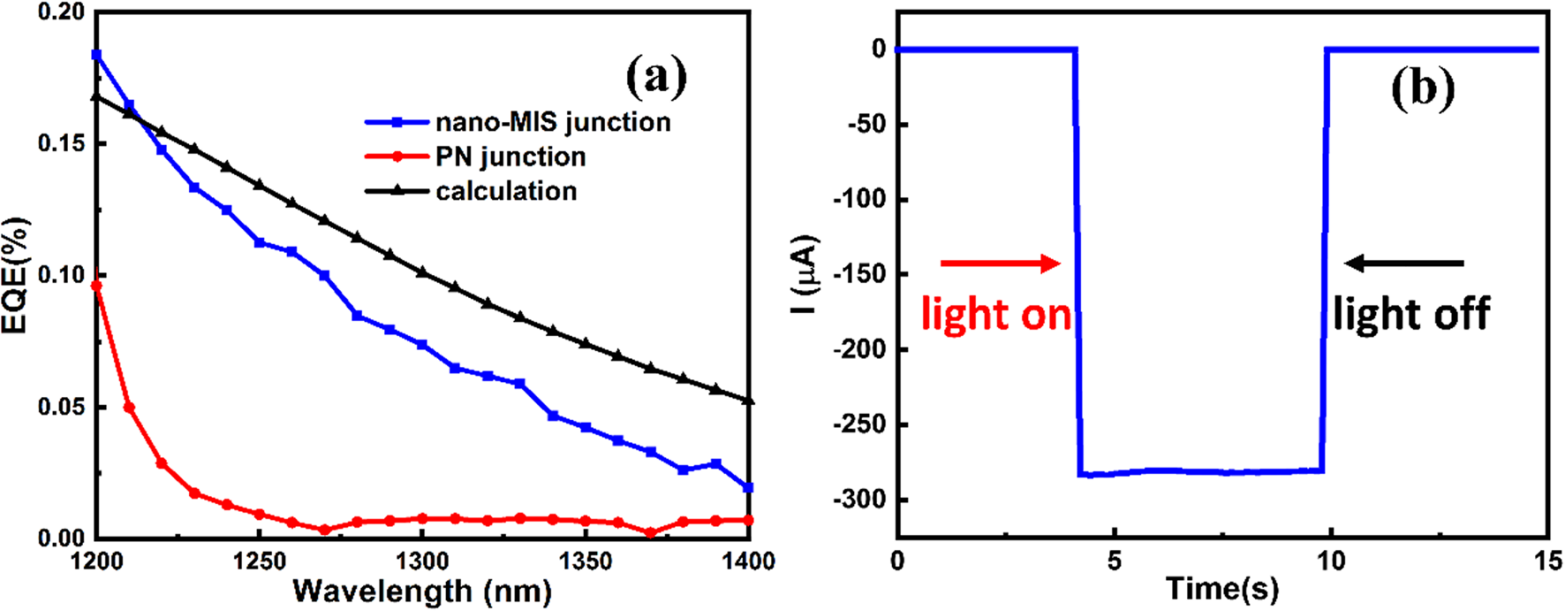
**a** Tested EQE curves of textured-Si/Al_2_O_3_/Au nano-MIS solar cell and PN junction Si solar cell in the wavelength range of 1200–1400 nm and calculated EQE based on Fowler equation. **b** Photoresponse of nano-MIS device at zero bias under 0.1 W/cm^2^ 1319 nm light illumination

Figure 8a gives the EQE curve of photovoltaic response between 1200 and 1400 nm for the best nano-MIS PV device. The EQE at 1319 nm was 0.06%. For comparison, the EQE of a commercial PN junction crystalline Si solar cell was also drawn. In 1200–1250 nm wavelength range, the EQE of the PN junction solar cell dropped sharply almost to zero, as it could hardly absorb the sub-bandgap NIR light. However, the EQE of the nano-MIS structure was > 0.1%, and decreased more slowly beyond 1250 nm. The internal photoemission yield can be described by simple Fowler equation [59]

1 $$Y(h\upsilon ) \approx \frac{1}{{8E_{F} }}\frac{{\left( {h\upsilon - \phi_{b} } \right)^{2} }}{h\upsilon },$$

where $\mathrm{h\upsilon }$ is energy of incident photon, ${\phi }_{b}$ is the barrier height in units of energy, and ${E}_{F}$ is the Fermi energy of the metal emitter. The EQE of device can then be approximated by $\mathrm{A}(\mathrm{h\upsilon })\cdot \mathrm{Y}(\mathrm{h\upsilon })$, where A is absorption of incident photon. Theoretical calculation result of EQE was also plotted in Fig. 8a. It can be noted that experimentally tested EQE was in good agreement with calculated EQE, both in absolute value and spectral line shape. Additionally, the extra J_SC_ gained from utilization of Si sub-bandgap NIR can be estimated by integral equation

2 $$J_{extra} = \int_{1200nm}^{1400nm} {q \cdot b_{S} (\lambda ) \cdot EQE(\lambda )d\lambda } ,$$

where q is electron charge, ${b}_{S}\left(\lambda \right)$ is photon flux density of solar spectrum. The result of extra J_SC_ calculated from tested EQE was ~ 0.01 mA/cm^2^. The photoresponse characteristic of nano-MIS device at zero bias was investigated in Fig. 8(b). The observed responsive current to 1319 nm light illumination was 282 μA, corresponding to the J_SC_ of device. Considering the dark current of 20 nA, it was proposed that our device could also function as a high-performance NIR photodetector which could operate at zero bias voltage, with responsivity (R) of 2.82 mA/W and detectivity (D^*^) at $3.5\times {10}^{10}$ cm × Hz^1/2^/W calculated as follows.

3 $$R = \frac{{I_{light} - I_{dark} }}{{P_{in} }},D^{ * } = \frac{R}{{\left( {2q \cdot J_{dark} } \right)^{1/2} }}$$

Compared to performance of NIR solar cell made by other materials such as PbS quantum dot working in the same wavelength range [60], the nano-MIS junction Si solar cell developed here had similar PV parameters of V_OC_ and FF, only the J_SC_ was one order of magnitude lower than their value, leading to our efficiency lower than theirs. Nonetheless, our device efficiency has reached the same level as the performance of TiO_2_ cells working in visible range. According to the theoretical analysis of efficiency limit by IPE mechanism [59, 61], the efficiency we obtained here was also reasonable, although it left room of promotion by, for example, improving the built-in electric field and further optimizing the generation, transportation and extraction of hot carriers [17]. Therefore, conversion efficiency close to 1% would be expected according to their simulation and this work could lay foundation for future researches on Si sub-bandgap NIR photovoltaic response.

## Conclusions

In summary, we investigated a sub-bandgap NIR (λ > 1100 nm) Si solar cell with nano-Au/Al_2_O_3_/n-Si MIS junctions, demonstrating the Si sub-bandgap NIR PV response that has not been exploited by Schottky junction solid-state device. It employed LSP-enhanced inner photoemission mechanism, and light trapping mechanism by textured surface. Under the illumination of 1319 nm light with intensity of 0.1 W/cm^2^, a best conversion efficiency achieved here was 0.034%, with open-circuit voltage of 0.21 V, short-circuit current density of 0.282 mA/cm^2^ and fill factor of 0.582, with the assistance of SiO_2_ and Al_2_O_3_ passivation. Our work is the first experimental demonstration of sub-bandgap NIR Si solar cell based on the MIS structure at room temperature. Such a Si-based plasmon-enhanced MIS photovoltaic device will be a promising strategy for application of Si sub-bandgap NIR photovoltaic response after further interfacial engineering.

## Supplementary Information

Supplementary file 1 (DOCX 558 KB)

## Data Availability

Raw data is available upon request from the corresponding author.

## Abbreviations

NIR: Near-infrared
PV: Photovoltaic
MIS: Metal–insulator–semiconductor
NP: Nanoparticle
IPE: Internal photoemission
PD: Photo-detection
LSP: Localized surface plasmons
ITO: Indium-tin-oxide
SEM: Scanning electron microscopy
EDS: Energy dispersive X-ray spectroscopy
EQE: External quantum efficiency
FDTD: Finite-difference time-domain
J_SC_: Short-circuit current density
V_OC_: Open-circuit voltage
FF: Fill factor

## Code Availability

Source code is available upon request from the corresponding author.

## References

[1] Nijs JF, Szlufcik J, Poortmans J, Sivoththaman S, Mertens RP (2001). Advanced cost-effective crystalline silicon solar cell technologies. Sol Energ Mat Sol C.

[2] Ribeyron PJ (2017). Crystalline silicon solar cells: Better than ever. Nat Energy.

[3] Haase F, Hollemann C, Schäfer S, Merkle A, Rienäcker M, Krügener J, Brendel R, Peibst R (2018). Laser contact openings for local poly-Si-metal contacts enabling 26.1%-efficient POLO-IBC solar cells. Sol Energ Mat Sol C.

[4] Green MA, Emery K, Hishikawa Y, Warta W, Dunlop ED, Levi DH, Ho Baillie AWY (2016). Solar cell efficiency tables (version 49). Prog in Photovolt Res App.

[5] W Yoon, Z Song, C Chen, D Scheiman, Y Yan. 21.1% efficient space perovskite/Si four-terminal tandem solar cells. In: IEEE 47th photovoltaic specialists conference (PVSC). 2020:1552–1556

[6] Yan X, Zhang C, Wang J, Zhang X, Ren X (2017). A high-efficiency Si nanowire array/perovskite hybrid solar cell. Nanoscale Res Lett.

[7] Chen D, Chen Y, Wang Z, Gong J, Verlinden PJ (2020). 24.58% total area efficiency of screen-printed, large area industrial silicon solar cells with the tunnel oxide passivated contacts (i-TOPCon) design. Sol Energ Mat Sol C.

[8] Wang LX, Zhou ZQ, Zhang TN, Chen X, Lu M (2016). High Fill factors of Si solar cells achieved by using an inverse connection between MOS and PN junctions. Nanoscale Res Lett.

[9] Shockley W, Queisser HJ (1961). Detailed balance limit of efficiency of p-n junction solar cells. J Appl Phys.

[10] Trupke T, Green MA, Würfel P (2002). Improving solar cell efficiencies by up-conversion of sub-band-gap light. J Appl Phys.

[11] Strümpel C, McCann M, Beaucarne G, Arkhipov V, Slaoui A, Švrček V, del Cañizo C, Tobias I (2007). Modifying the solar spectrum to enhance silicon solar cell efficiency—an overview of available materials. Sol Energ Mat Sol C.

[12] Badescu V, Badescu AM (2009). Improved model for solar cells with up-conversion of low-energy photons. Renew Energy.

[13] Hayat A, Ginzburg P, Orenstein M (2008). Infrared single-photon detection by two-photon absorption in silicon. Phys Rev B.

[14] Richards BS, Shalav A (2007). Enhancing the near-infrared spectral response of silicon optoelectronic devices via up-conversion. IEEE Trans Electron Dev.

[15] García De Arquer FP, Konstantatos G (2015). Metal-insulator-semiconductor heterostructures for plasmonic hot-carrier optoelectronics. Opt Express.

[16] Clavero C (2014). Plasmon-induced hot-electron generation at nanoparticle/metal-oxide interfaces for photovoltaic and photocatalytic devices. Nat Photonics.

[17] Zhu YS, Xu HX, Yu P, Wang ZM (2021). Engineering plasmonic hot carrier dynamics toward efficient photodetection. Appl Phys Rev.

[18] Desiatov B, Goykhman I, Mazurski N, Shappir J, Khurgin JB, Levy U (2015). Plasmonic enhanced silicon pyramids for internal photoemission Schottky detectors in the near-infrared regime. Optica.

[19] Casalino M, Coppola G, De La Rue RM, Logan DF (2016). State-of-the-art all-silicon sub-bandgap photodetectors at telecom and datacom wavelengths. Laser Photonics Rev.

[20] Hu F, Dai X, Zhou Z, Kong X, Sun S, Zhang R, Wang S, Lu M, Sun J (2019). Black silicon Schottky photodetector in sub-bandgap near-infrared regime. Opt Express.

[21] Hu F, Wu L, Dai XY, Li S, Lu M, Sun J (2021). Achieving high-responsivity near-infrared detection at room temperature by nano-Schottky junction arrays via a black silicon/platinum contact approach. Photonics Res.

[22] García De Arquer FP, Mihi A, Kufer D, Konstantatos G (2013). Photoelectric energy conversion of plasmon-generated hot carriers in metal–insulator–semiconductor structures. ACS Nano.

[23] Takahashi Y, Tatsuma T (2011). Solid state photovoltaic cells based on localized surface plasmon-induced charge separation. Appl Phys Lett.

[24] Qi ZY, Zhai YS, Wen L, Wang Q, Chen Q, Iqbal S, Chen GD, Xu J, Tu Y (2017). Au nanoparticle-decorated silicon pyramids for plasmon-enhanced hot electron near-infrared photodetection. Nanotechnology.

[25] Wen L, Chen YF, Liu WW, Su Q, Grant J, Qi ZY, Wang QL, Chen Q (2017). Enhanced photoelectric and photothermal responses on silicon platform by plasmonic absorber and Omni-Schottky junction. Laser Photonics Rev.

[26] Wen L, Liang L, Yang XG, Liu Z, Li BJ, Chen Q (2019). Multiband and ultrahigh figure-of-merit nanoplasmonic sensing with direct electrical readout in Au-Si nanojunctions. ACS Nano.

[27] ChuHsuan L, Wee LC (2010). Metal-insulator-semiconductor photodetectors. Sensors.

[28] Padmanabhan R, Sorias O, Eyal O, Mikhelashvili V, Orenstein M, Eisenstein G (2017). Responsivity enhancement of metal-insulator-semiconductor photodetectors on silicon-on-insulator substrates by plasmonic nanoantennas. IEEE Trans Nanotechnol.

[29] Lee JY, Glunz SW (2006). Investigation of various surface passivation schemes for silicon solar cells. Sol Energ Mat Sol C.

[30] Chen YL, Zhong SH, Tan M, Shen WZ (2016). SiO_2_ passivation layer grown by liquid phase deposition for silicon solar cell application. Front Energy.

[31] García-Valenzuela JA, Rivera R, Morales-Vilches AB, Gerling LG, Caballero A, Asensi JM, Voz C, Bertomeu J, Andreu J (2016). Main properties of Al_2_O_3_ thin films deposited by magnetron sputtering of an Al_2_O_3_ ceramic target at different radio-frequency power and argon pressure and their passivation effect on p-type c-Si wafers. Thin Solid Films.

[32] Hoex B, Gielis JJH, Sanden VDMC, Kessels WMM (2008). On the c-Si surface passivation mechanism by the negative-charge-dielectric Al_2_O_3_. J Appl Phys.

[33] Schmidt J, Merkle A, Brendel R, Hoex B, de Sanden MCMV, Kessels WMM (2008). Surface passivation of high-efficiency silicon solar cells by atomic-layer-deposited Al_2_O_3_. Prog Photovolt Res Appl.

[34] Qiu Y, Wang LX, Hao HC, Shi W, Lu M (2015). A synergetic effect of surface texture and field-effect passivations on improving si solar cell performance. Phys E Low Dimens Syst Nanostructures.

[35] Serrano A, de la Rodrígueza Fuente O, García MA (2010). Extended and localized surface plasmons in annealed Au films on glass substrates. J Appl Phys.

[36] Spitzer WG, Fan HY (1957). Infrared absorption in *n*-Type Silicon. Phys Rev.

[37] Tayagaki T, Kishimoto Y, Hoshi Y, Usami N (2015). Absorption enhancement in nanotextured solar cells with Ge/Si heterostructures. Jpn J Appl Phys.

[38] Tayagaki T, Furuta D, Aonuma O, Takahashi I, Hoshi Y, Kurokawa Y, Usami N (2017). Optical characterization of double-side-textured silicon wafer based on photonic nanostructures for thin-wafer crystalline silicon solar cells. Jpn J Appl Phys.

[39] SH Zaidi, DS Ruby, K Dezetter, JM Gee. Enhanced near IR absorption in random, RIE-textured silicon solar cells: the role of surface profiles. In: IEEE photovoltaic specialists conference. 2002:142–145.

[40] Yatsui T (2019). Recent improvement of silicon absorption in opto-electric devices. Opto Electron Adv.

[41] Sobhani A, Knight MW, Wang Y, Zheng B, King NS, Brown LV, Fang Z, Nordlander P, Halas NJ (2013). Narrowband photodetection in the near-infrared with a plasmon-induced hot electron device. Nat Commun.

[42] Knight MW, Sobhani H, Nordlander P, Halas NJ (2011). Photodetection with active optical antennas. Science.

[43] Nishijima Y, Ueno K, Yokota Y, Murakoshi K, Misawa H (2010). Plasmon-assisted photocurrent generation from visible to near-infrared wavelength using a Au-Nanorods/TiO_2_ electrode. J Phys Chem Lett.

[44] Losurdo M, Giangregorio MM, Bianco GV, Sacchetti A, Capezzuto P, Bruno G (2009). Enhanced absorption in Au nanoparticles/a-Si:H/c-Si heterojunction solar cells exploiting Au surface plasmon resonance. Sol Energ Mat Sol C.

[45] Fang Y, Jiao Y, Xiong K, Ogier R, Yang Z, Gao S, Dahlin AB, Käll M (2015). Plasmon enhanced internal photoemission in antenna-spacer-mirror based Au/TiO_2_ nanostructures. Nano Lett.

[46] Zhao JP, Lu M, Chen ZY, Rabalais JW (2002). Surface-plasmon-resonance-induced absorption of a metal–oxide nanoparticle composite. Appl Phys Lett.

[47] Zhou ZQ, Qiu Y, Shi W, Sun T, Li YL, Lu M (2014). Surface plasmons on Ag clusters induced via ultrasonic and thermal treatments and the enhancement of Si nanocrystal light emission. Phys E.

[48] Nazirzadeh MA, Atar FB, Turgut BB, Okyay AK (2014). Random sized plasmonic nanoantennas on Silicon for low-cost broad-band near-infrared photodetection. Sci Rep.

[49] Lee YK, Lee H, Lee C, Hwang E, Park JY (2016). Hot-electron-based solar energy conversion withmetal–semiconductor nanodiodes. J Phys Condens Matter.

[50] Tang H, Chen C-J, Huang Z, Bright J, Meng G, Liu R-S, Nianqiang Wu (2020). Plasmonic hot electrons for sensing, photodetection, and solar energy applications: a perspective. J Chem Phys.

[51] Sze SM, Moll JL, Sugano T (1964). Range-energy relation of hot electrons in gold. Solid State Electron.

[52] Crowell CR, Sze SM (1965). Ballistic mean free path measurements of hot electrons in Au films. Phys Rev Lett.

[53] Voisin C, Del Fatti N, Christofilos D, Vallée F (2001). Ultrafast electron dynamics and optical nonlinearities in metal nanoparticles. J Phys Chem B.

[54] Li X, Deng Z, Ma Z, Jiang Y, Du C, Jia H, Wang W, Chen H (2022). Demonstration of SWIR silicon-based photodetection by using Thin ITO/Au/Au nanoparticles/n-Si structure. Sensors.

[55] Ponpon JP, Siffert P (1976). Open-circuit voltage of MIS silicon solar cells. J Appl Phys.

[56] Nelson J (2018). The physics of solar cells.

[57] Wronski CR, Abeles B, Cody GD, Tiedje T (1980). Internal photoemission in hydrogenated amorphous-Si films. Appl Phys Lett.

[58] Joseph ML, Matt L, Matthew CB, Qing S, Matthew OR, Randy JE, Arthur JN (2008). Schottky solar cells based on colloidal nanocrystal films. Nano Lett.

[59] Leenheer AJ, Narang P, Lewis NS, Atwater HA (2014). Solar energy conversion via hot electron internal photoemission in metallic nanostructures: efficiency estimates. J Appl Phys.

[60] Bi Y, Pradhan S, Gupta S, Akgul MZ, Stavrinadis A, Konstantatos G (2018). Infrared solution-processed quantum dot solar cells reaching external quantum efficiency of 80% at 135 µm and *J*_sc_ in excess of 34 mA cm^−2^. Adv Mat.

[61] White TP, Catchpole KR (2012). Plasmon-enhanced internal photoemission for photovoltaics: theoretical efficiency limits. Appl Phys Lett.

