# Supplementary material for: Sub-bandgap near-infrared photovoltaic response in Au/Al2O3/n-Si metal–insulator–semiconductor structure by plasmon-enhanced internal photoemission
Source: Discov Nano. 2023 Mar 7;18(1):33. doi: 10.1186/s11671-023-03818-4 (PMC9992651; doi:10.1186/s11671-023-03818-4)
Supplement: Supplementary file 1 — (DOCX 558 KB) [file 11671_2023_3818_MOESM1_ESM.docx]

**Supplementary Information**

**Sub-bandgap Near-infrared Photovoltaic Response in Au/Al_2_O_3_/n-Si Metal-Insulator-Semiconductor Structure by Plasmon-enhanced Internal Photoemission**

Xiyuan Dai^1^, Li Wu^1^, Liang Yu^1^, Zhiyuan Yu^1^, Fengyang Ma^1^,Yuchen Zhang^1^, Yanru Yang^1^, Jian Sun^1,2*^, Ming Lu^1,2*^

^1^Department of Optical Science and Engineering, and Shanghai Ultra-Precision Optical Manufacturing Engineering Center, Fudan University, Shanghai 200433, China

^2^Yiwu Research Institute of Fudan University, Yiwu 322000, Zhejiang, China

^*^jsun@fudan.edu.cn

^*^minglu55@fudan.edu.cn


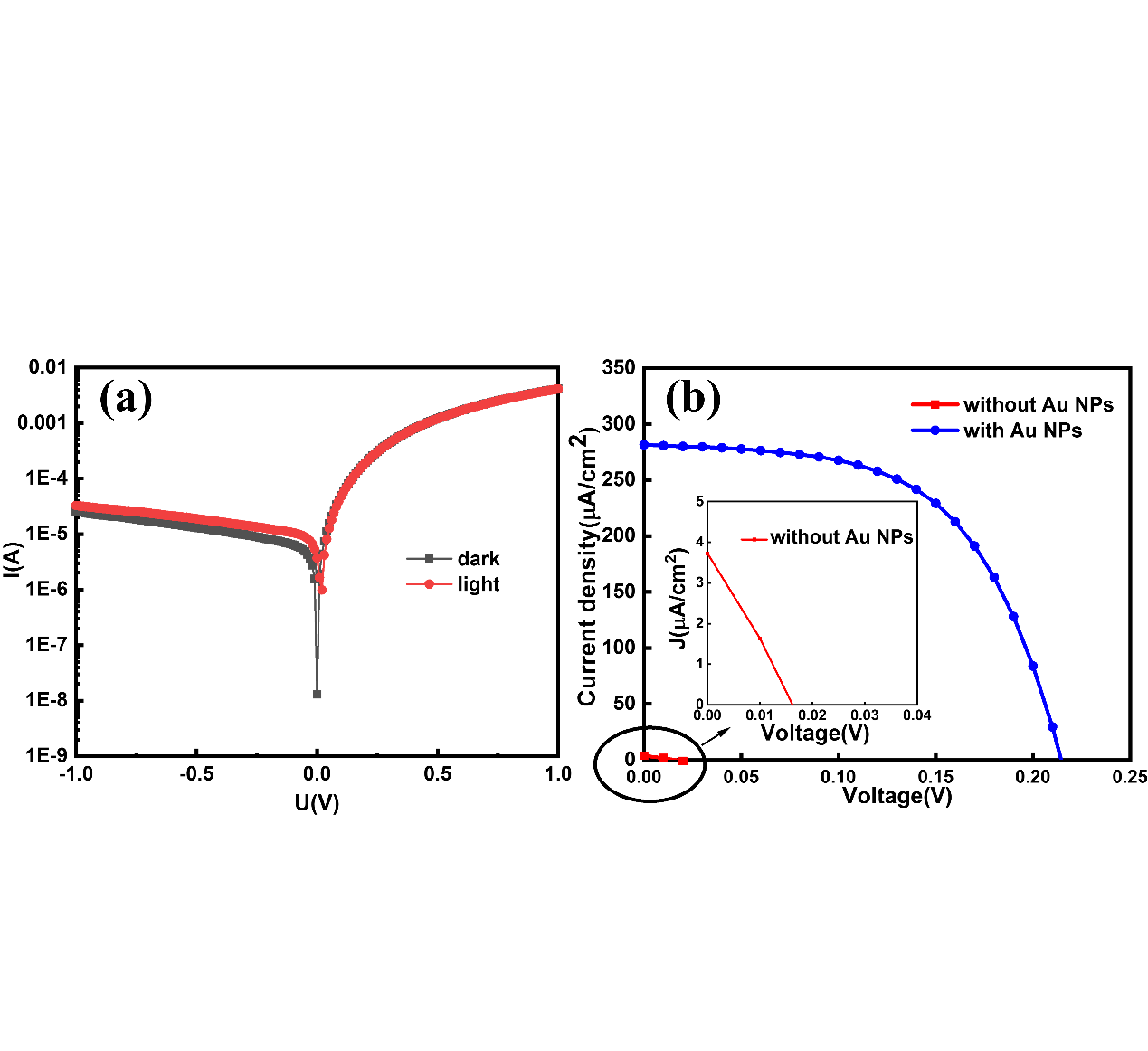


**Figure S1** **(a)** I-V characteristics of ITO/Al_2_O_3_/n-Si structure device without Au NPs under dark and 1319 nm light illumination. **(b)** Photovoltaic response of ITO/Al_2_O_3_/n-Si and Au/Al_2_O_3_/n-Si nano-MIS solar cell under 1319 nm light

For ITO/Al_2_O_3_/n-Si structure device, despite the NIR absorption of textured Si, almost no photovoltaic response was found, with J_SC_ of only ~4 $\mu A$/cm^2^ and V_OC_ of ~0.02V which were not comparable with Au/Al_2_O_3_/n-Si device. This could be attributed to the fact that NIR absorption of textured n-Si occurred mainly in bulk Si and the generated electrons and holes could not be separated effectively by the electric field located at ITO/n-Si interface. It could be concluded that Au NPs were the main absorber and determinant factor for the photoresponse of 1319 nm light.


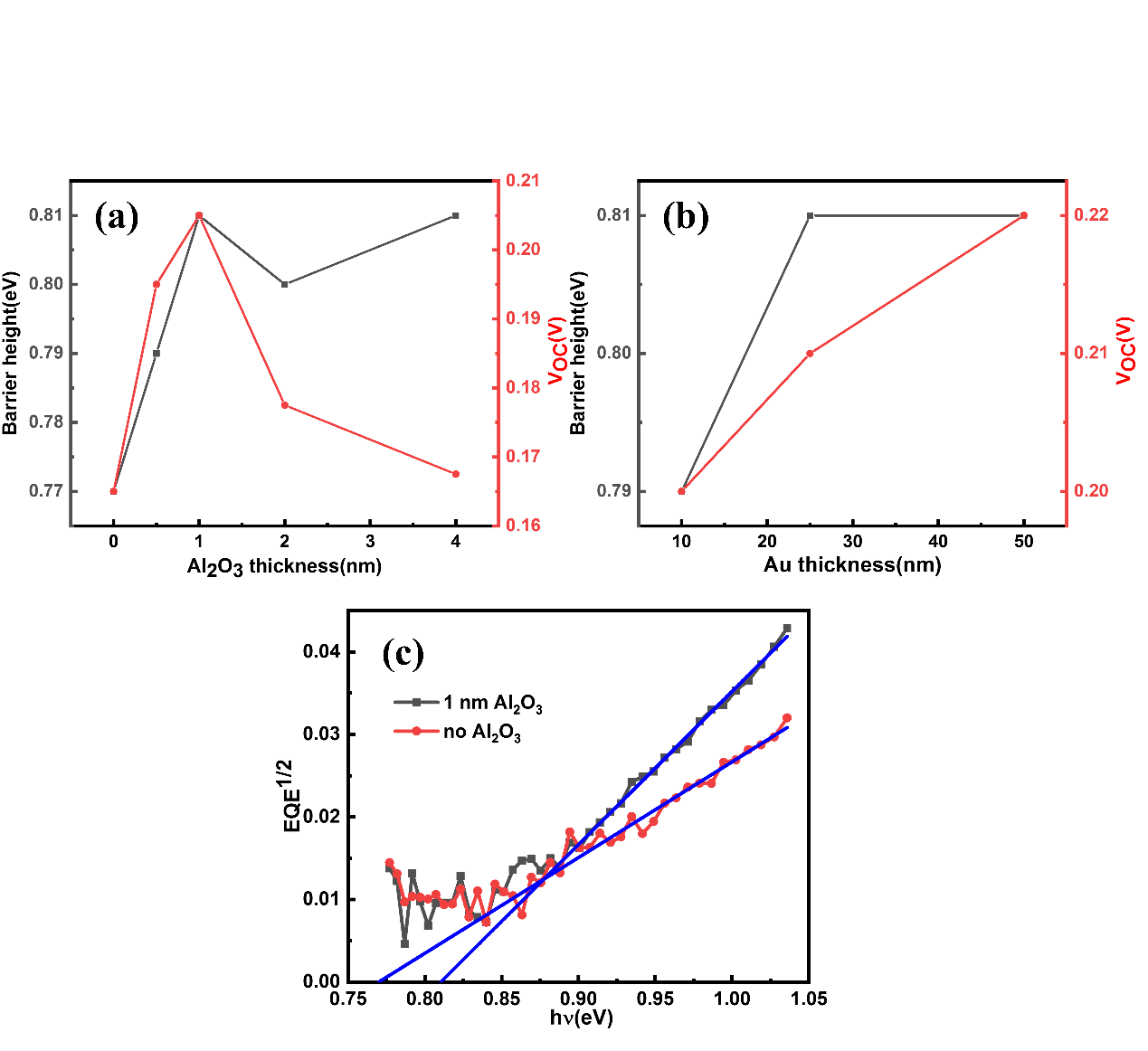


**Figure S2** The barrier height of Au/Al_2_O_3_/n-Si structure for **(a)** different Al_2_O_3_ thickness with 25 nm Au and **(b)** different Au NPs sizes with 1 nm Al_2_O_3_, along with the V_OC_ of device. **(c)** The fitting of square root of EQE versus photon energy to obtain the barrier height by x-intercept

Within 1 nm Al_2_O_3_ thickness, the V_OC_ of device increased in accordance with the barrier height when it was raised from 0.77 eV to 0.81 eV. When the Al_2_O_3_ thickness was larger than 1 nm, the barrier remained high and the lower V_OC_ could be attributed to the decrease of J_SC_. After annealing thicker Au film, the formation of larger area of MIS junction could induce higher barrier, consistent with better V_OC_ in device.


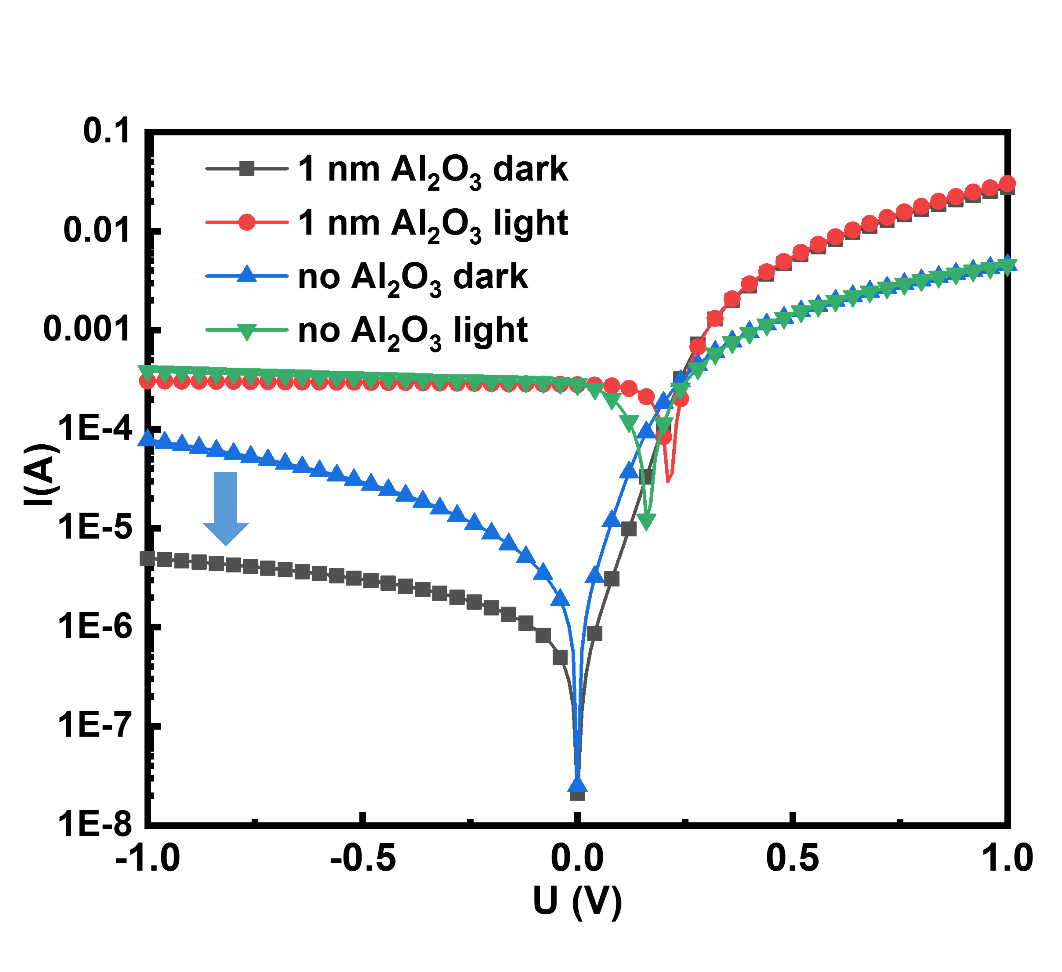


**Figure S3** The I-V characteristics of Au/Si structure device and nano-MIS device with 1 nm Al_2_O_3_ under dark and 1319 nm light condition

Since the saturation current density at reverse bias can be expressed as $J_{S}=A^{*}T^{2}exp(-\frac{e\phi_{b}}{kT})$, where A* is the Richardson constant, the lower saturation current after adding Al_2_O_3_ can verify the increment of barrier height.


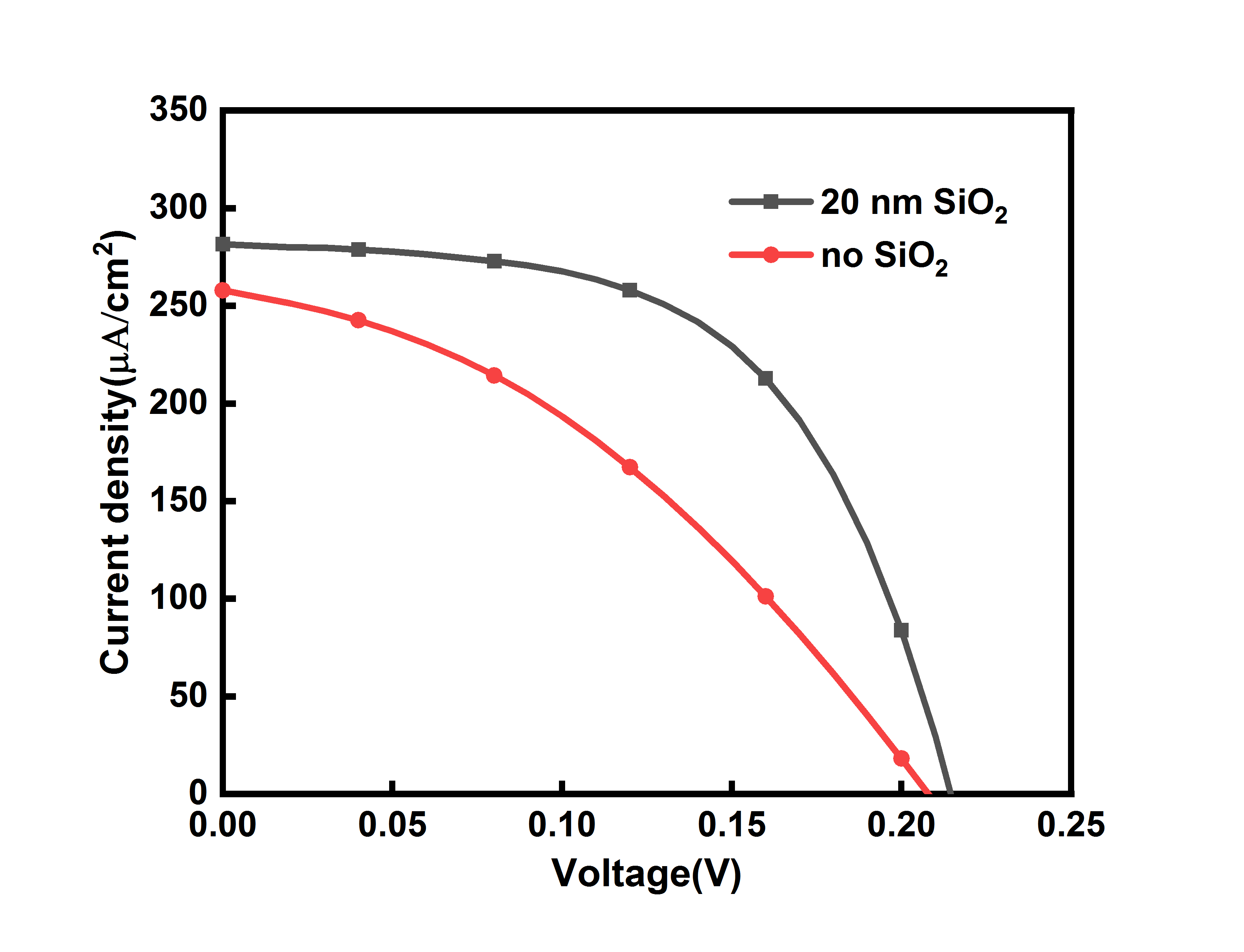


**Figure S4** The J-V characteristics of Au/Al_2_O_3_/n-Si nano-MIS device with or without SiO_2_ on the rear surface under 1319 nm light illumination

By adopting SiO_2_, the device could have relatively higher J_SC_, V_OC_ and FF, which verified the diminishment of surface defects and field effect passivation by SiO_2_ layer.


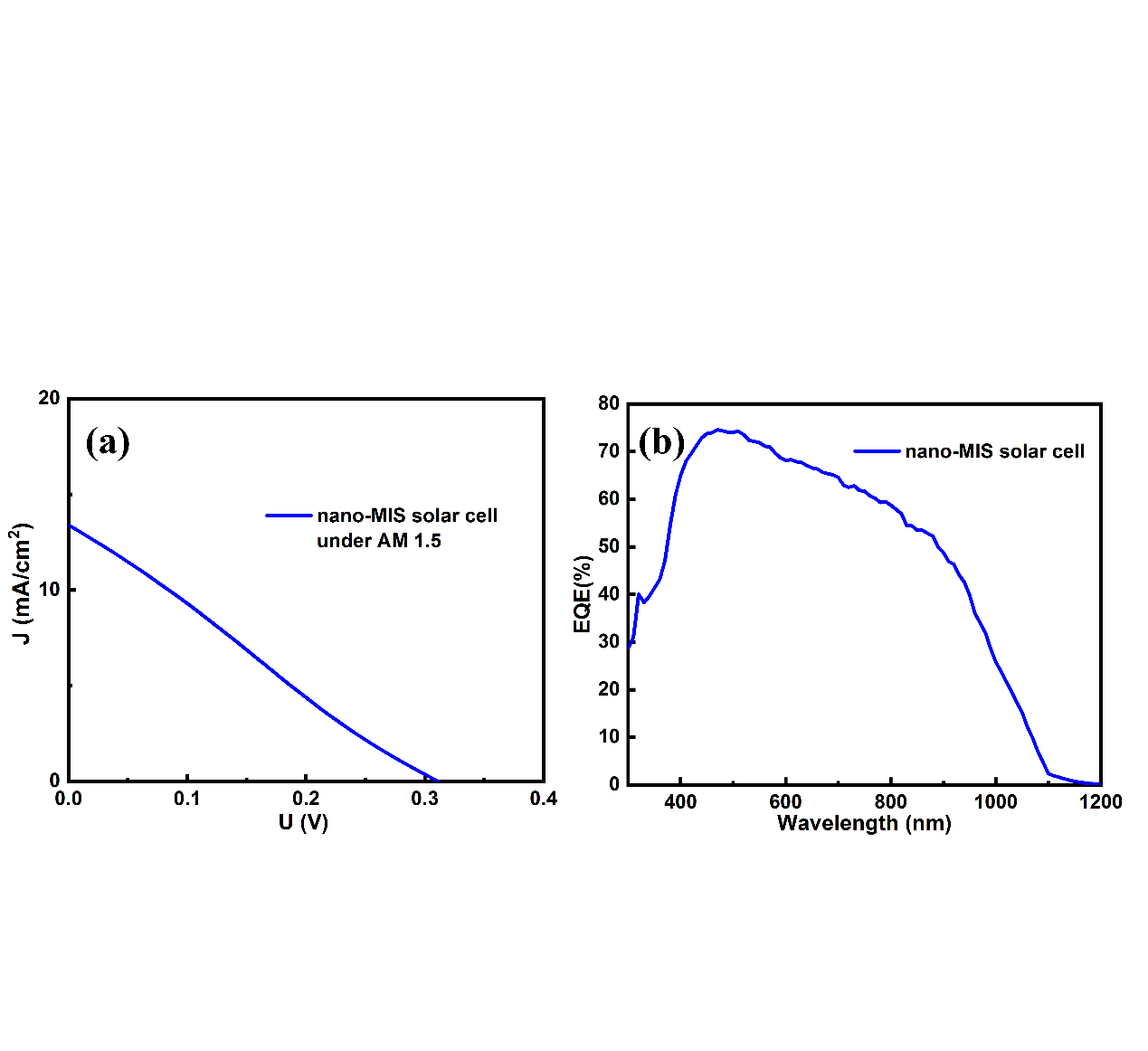


**Figure S5** **(a)** The J-V characteristics of the nano-MIS solar cell proposed in this work under AM 1.5 solar simulator. **(b)** EQE of the nano-MIS solar cell in 300-1200 nm

The conversion efficiency was 1.03% with V_OC_ of 0.31 V, J_SC_ of 13.38 mA/cm^2^ and FF of 24.83% under AM 1.5. Though the efficiency and EQE was not high, our device was intended to utilize Si sub-bandgap NIR light and could serve as the bottom subcell in stack configuration of solar cell. After enhancing the PV response to sub-bandgap NIR, our device could be connected with PN junction solar cell by parallel stack configuration, broadening the spectral response of Si solar cells in an all-silicon way.


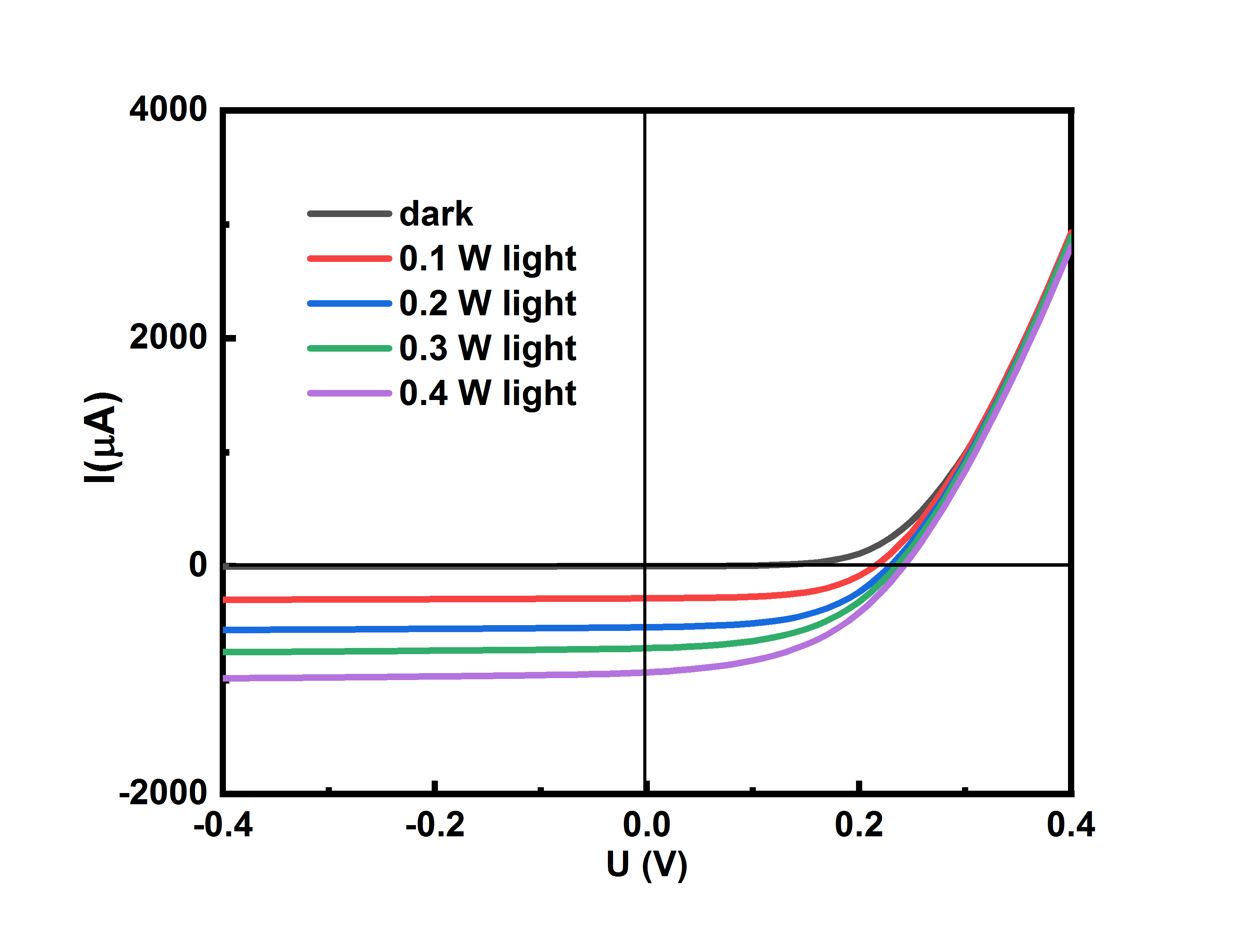


**Figure S6** The I-V characteristics of the nano-MIS solar cell under different incident light intensity of 1319 nm

With the increasing light intensity, the J_SC_ increased linearly and V_OC_ grew in a logarithmic way, showing the general solar cell properties.
